# Navigating Sarcopenia Risks in GLP-1RA Therapy for Advanced Heart Failure

**DOI:** 10.3390/biomedicines13051108

**Published:** 2025-05-02

**Authors:** Winston Wang, Danielle Green, Ramzi Ibrahim, Mahmoud Abdelnabi, Hoang Nhat Pham, Beani Forst, Mohamed Allam, Patrick Sarkis, George Bcharah, Juan Farina, Chadi Ayoub, Dan Sorajja, Reza Arsanjani

**Affiliations:** 1 Department of Medicine, Mayo Clinic, Phoenix, AZ 85054, USA; wang.winston@mayo.edu (W.W.); green.danielle@mayo.edu (D.G.); 2 Department of Cardiovascular Medicine, Mayo Clinic, Scottsdale, AZ 85259, USA; abdelnabi.mahmoud@mayo.edu (M.A.); forst.beani@mayo.edu (B.F.); allam.mohamed@mayo.edu (M.A.); sarkis.patrick@mayo.edu (P.S.); farina.juanmaria@mayo.edu (J.F.); ayoub.chadi@mayo.edu (C.A.); sorajja.dan@mayo.edu (D.S.); arsanjani.reza@mayo.edu (R.A.); 3 Department of Medicine, University of Arizona, Tucson, AZ 85724, USA; npham917@arizona.edu; 4 Mayo Clinic Alix School of Medicine, Phoenix, AZ 85054, USA; bcharah.george@mayo.edu

**Keywords:** heart failure, frailty, sarcopenia, GLP-1RA

## Abstract

Cardiac cachexia (CC) is a severe complication of advanced heart failure (HF), characterized by involuntary weight loss and muscle wasting, leading to poor outcomes and higher mortality. Despite its severity, CC remains under-recognized and undertreated, lacking targeted therapies specifically addressing its pathophysiology. Glucagon-like peptide-1 receptor agonists (GLP-1RAs), though beneficial in reducing cardiovascular risk in patients with HF, may exacerbate muscle wasting in cachectic patients, necessitating further investigation. Non-pharmacological strategies, including tailored nutritional support and exercise programs, have shown positive effects on body composition and quality of life in patients with CC. However, there remains a gap in recommendations tailored to preventive strategies and pharmacologic therapies for patients with CC and concomitant GLP-1RA use. This review highlights the multifactorial mechanisms underlying CC and current and emerging therapeutic approaches for mitigating HF-related sarcopenia while on GLP-1RAs.

## 1. Introduction

Heart failure (HF) is a prevalent condition affecting over six million individuals in the United States, with its burden expected to rise due to the increasing prevalence of risk factors such as obesity, hypertension, and diabetes [1]. Obesity is especially closely associated with the pathogenesis and progression of HF with preserved ejection fraction (HFpEF), and over 80% of patients with HFpEF also have comorbid obesity [2,3]. Over the last decade, significant advancements have been made in the medical management of HF, expanding therapeutic options and improving outcomes. The benefits of glucagon-like peptide-1 receptor agonists (GLP-1RAs) in cardiovascular outcomes are well-established, as demonstrated by multiple cardiovascular outcome trials (CVOTs), which show reductions in death from cardiovascular causes, non-fatal stroke, and non-fatal myocardial infarction [4]. Ongoing research continues to expand our understanding of their clinical utility, leading to broader guideline-approved indications.

Initially approved in 2005 for the management of type 2 diabetes mellitus (DM2), short-acting GLP-1RAs were recognized for their ability to lower plasma glucose via glucose-dependent insulinotropic effects, inhibition of excessive glucagon secretion, and delayed gastric emptying [5]. By 2014, evidence from phase 3 clinical trials under the SCALE program supported the use of liraglutide combined with lifestyle interventions for weight management in individuals with obesity or elevated BMI and comorbidities [6]. These studies demonstrated significant weight loss and cardiovascular benefits, including improvements in lipid profiles [6]. Their use was catapulted into public knowledge with two landmark trials for weight loss in non-diabetic patients. In a 56-week, double-blind, placebo-controlled trial involving 3731 non-diabetic patients with increased BMI, those receiving liraglutide achieved a mean weight loss of 8.4 kg (~8% body weight) compared to 2.4 kg (~2.6%) in the placebo group [7]. Investigations into other GLP1 receptor agonists were pursued, and both semaglutide and tirzepatide were evaluated for weight loss including nondiabetic patients. In the STEP 1 trial, a 68-week randomized controlled trial involving semaglutide in non-diabetic patients with increased BMI, patients experienced a mean weight loss of 15.3 kg (~14.9%) compared to 2.6 kg (~2.4%) with placebo [8]. In the SURMOUNT 1 trial, a 72-week randomized control trial in patients with elevated BMI, this time with tirzepatide, patients also experienced significant weight loss of about 20% at higher dosages compared to the placebo [9].

Cardiac cachexia (CC) is a multifactorial syndrome characterized by involuntary weight loss due to tissue breakdown as a consequence of chronic disease [10]. CC has been shown to share multiple common pathways with various disease entities, particularly as malnutrition is involved in a common pathogenetic chain with CC [11]. Specifically, CC arises in the setting of chronic HF, with an estimated prevalence of 5–15% among patients with advanced HF [10]. Its pathophysiology involves a complex interplay of neurohormonal changes and inflammatory cytokines, both of which drive catabolic processes. Additional contributors include protein degradation, muscle wasting, and gastrointestinal dysfunction, all of which are exacerbated by decreased cardiac output (CO). The activation of the sympathetic nervous system (SNS) in this context diverts blood flow away from the gastrointestinal tract, while venous congestion leads to bowel wall edema and nutrient malabsorption [10].

Patients with CC frequently experience sarcopenia, which is closely linked to frailty syndrome. Both conditions share overlapping pathophysiological mechanisms, resulting in increased morbidity and mortality. Frailty syndrome, which is diagnosed in over 50% of patients with HF, independently predicts higher rates of hospital readmission, early disability, and long-term mortality [12,13]. A 2023 retrospective analysis of 2,360,307 hospitalized HF patients highlighted the severity of CC, finding that it was associated with significantly higher inpatient mortality (odds ratio: 3.01; 95% CI: 2.88–3.15; *p* < 0.001) [14].

With the profound impact of CC on morbidity and mortality in patients with HF, there is a critical need to understand and explore the impact of GLP-1RAs on HF outcomes particularly related to CC, with concerns for the risk of worsening sarcopenia. Recent trials have revealed the remarkable impact of GLP-1RAs in the HF population and while the expected utilization rates of this pharmacological agent are to increase in coming years, there is a lack of synthesized data on recommendations to promote sarcopenia-mitigating protocols. Therefore, this review explores the types of body composition compartments that are susceptible to weight loss and the literature behind preventive strategies to mitigate sarcopenia from GLP-1RAs.

## 2. Weight Loss with GLP-1RAs

While the weight loss induced by GLP-1RAs is clinically significant, their impact on muscle mass warrants further scrutiny, particularly in vulnerable populations with advanced HF. In addition, there has been conflicting evidence with regard to the impact of obesity on adverse effects in heart failure patients, with some studies showing a signal for improved outcomes in patients with comorbid heart failure and obesity, also known as the obesity survival paradox [15]. Specifically in patients with heart failure with preserved ejection fraction, the STEP-HFpEF trial showed promise in reducing heart failure-related symptoms as measured by the Kansas City Cardiomyopathy Questionnaire Clinical Summary Score (KCCQ-CSS) in patients with obesity-related heart failure. They found statistically significant weight loss in the GLP1RA group, with a mean between-group difference in body weight of −8.4% (95% CI −9.2 to −7.5; *p* < 0.0001) [16]. This weight loss was associated with significant improvement in functional status as measured by the six-minute walk distance and KCCQ-CSS. Importantly, these clinical improvements were associated with a decrease in cardiac disorder events compared to the placebo group (7 [2.7%] vs. 30 [11.3%] in the placebo group; *p* < 0.001) [16]. In addition, the SUMMIT trial utilized tirzepatide, a dual GIP and GLP1 receptor agonist, in patients with HFpEF [17]. They demonstrated that tirzepatide led to significant weight loss, improved cardiovascular outcomes, decreased heart failure exacerbations, and improved KCCQ-CSS. However, the FIGHT trial, a smaller trial of 300 patients with HFrEF, both with and without diabetes or obesity, studied the use of liraglutide in HFrEF [18]. They found that it was also effective in weight loss, with a treatment difference −4.10 lbs (95% CI −7.94 to −0.25; *p* = 0.0367); however, there was a non-statistically significant trend towards increased hospitalization rates in the liraglutide treatment group. They theorized that there exists a discordance in outcomes with weight loss between patients with low BMI versus patients with elevated BMI, and that patients with HFrEF with low BMI potentially are harmed by further weight loss [18].

There are several proposed mechanisms against obesity survival paradox and supporting the benefits of weight loss in heart failure, including distinguishing intentional vs. unintentional weight loss [3], earlier presentation in obese patients [19], and BMI as an inaccurate measure of body composition, especially in heart failure [20]. One trial compared the effects of caloric restriction and exercise in patients with chronic HFpEF, and showed that the treatment arms (exercise, diet, and exercise + diet) were able to achieve improvements in weight loss, lean body mass and maximum VO2 without any significant changes in the frequency of adverse effects [3]. Another trial utilizing subgroup analyses of the PARADIGM-HF trial demonstrated that with newer anthropometric measures beyond BMI, there was no evidence of increased survival with obesity. Specifically, they compared BMI to several other indices including waist–height ratio, a weight-agnostic measure of obseity, and found that the increased risk of mortality seen with low BMI was eliminated with the newer indices, when adjusted for conventional risk variables [20]. Moreover, they found that with the newer, weight-agnostic indices, there was in fact a significantly higher risk for HF exacerbations in the upper quintile, which was less apparent with BMI. This discrepancy could be explained by elimination of confounding from fluid retention and unintentional weight loss. Finally, a recent meta-analysis of over 5000 patients suggested that obesity may be associated with better prognosis, but not causal, due to being also associated with less advanced heart failure and fewer comorbidities [21]. The study included both patients with HFpEF and patients with HFrEF and used multivariate Cox regression to control for comorbid conditions, biomarkers such as NT-proBNP, and medication use. They found that although there was a significantly increased risk for all-cause mortality and heart failure hospitalizations with a BMI of <25, this risk was eliminated with age < 75 and controlling for comorbidities [21].

Given the complex interplay between obesity and heart failure, the assessment of body composition has also been utilized to further characterize the types of weight loss induced by GLP1RAs. Body composition includes several compartments: fat mass (FM), fat-free mass (FFM), lean mass (LM), skeletal muscle mass (SMM), and adipose tissue (AT) [22,23]. LM comprises non-fat tissues such as muscles and organs (excluding bone), while SMM specifically refers to skeletal muscle. FM accounts for all lipid content in the body, and FFM encompasses all non-fat components, including bone, lean tissues, and water [22]. Recent studies provide insights into body composition changes associated with GLP-1RAs (Table 1). For example, in the STEP-1 trial, a randomized control trial involving 1961 nondiabetic patients with increased BMI, semaglutide (2.4 mg/week for 68 weeks) reduced FM by 24.7% and LM by 13.9% [8]. Similarly, in a subgroup analysis of the SUSTAIN-8 trial, which examined semaglutide in diabetic patients on metformin, semaglutide was shown to have numerical improvements in body composition, such as a reduction in both FM by 10.2% and LM by 4.5%. They also noted a corresponding increase in proportion of lean mass, up 1.2% from the baseline [24]. The SURMOUNT-1 trial, a similar randomized control trial with 2359 nondiabetic patients with increased BMI, showed that tirzepatide, a dual GLP-1 and GIP receptor agonist, administered weekly for 72 weeks, resulted in a 33.9% reduction in FM and a 10.9% reduction in LM [9]. While reductions in FM are therapeutically significant, the concurrent loss of LM, primarily muscle, is concerning, as it can contribute to frailty, increased risk of falls, and hospitalization [25,26]. The extent of LM loss (~6.5 kg over ~70 weeks) observed in patients on GLP-1RAs is comparable to the muscle decline experienced over a decade of aging [27,28,29].

## 3. Counteracting Muscle Loss During GLP-1RA Use

### 3.1. Nutritional Support

Nutritional support plays a critical role in the management of CC and sarcopenia. Dietary supplementation with fish oil containing polyunsaturated fatty acids (PUFAs), including eicosapentaenoic acid (EPA) and docosahexaenoic acid (DHA), have demonstrated potential benefits in patients with chronic HF. These include improved increased exercise tolerance and decreased circulating cytokine levels [30,31]. Similar interventions have been shown to yield positive outcomes in CC specifically [32,33], including high-calorie and high-protein supplements [34], PUFAs [35], and L-alanyl-L-glutamine [36].

One of the earliest interventional studies in CC, conducted in 2010, was a randomized, double-blind trial examining the effects of a high-calorie, high-protein oral nutritional supplement in patients with chronic HF and cachexia (Table 2). This study demonstrated significant improvements in body weight, body composition, and quality of life [34]. Over 6 weeks, patients gained an average of 2.0 ± 1.7 kg, which increased to 2.3 ± 3.1 kg at 18 weeks. Additionally, there were reductions in serum levels of tumor necrosis factor-α and improvements in quality of life. A more recent study investigated the effects of oral supplementation composed of slow-release carbohydrates, fiber, and n-3 and n-6 fatty acids in 38 patients with HF. Among them, 66% met the criteria for sarcopenia and 24% for malnutrition [35]. After six months of nutritional intervention, patients experienced increases in body weight and fat percentage, alongside reductions in inflammatory markers. Functional improvements, measured by the up-and-go test, were also improved.

One randomized, double-blind, placebo-controlled trial evaluated the effects of daily supplementation with a combination of L-alanyl-L-glutamine (8 g/day) and PUFAs (6.5 g/day) over three months in 31 patients with chronic HF [36]. Although there were no significant changes in muscle function, molecular markers from muscle biopsies, exercise performance, or echocardiographic parameters, the treatment group showed increases in lean body mass and improvements in quality of life.

Additional nutritional supplements, including beta-hydroxy-beta-methylbutyrate [37], branched-chain amino acids [38], and leucine [39], have shown promise in both human and animal models. While further research is needed, these interventions hold potential for improving outcomes in CC and sarcopenia by promoting muscle preservation and overall metabolic health. Interventions that combine adequate calories, high-quality protein, and PUFAs have demonstrated potential to improve body composition, reduce inflammation, and enhance quality of life. Nonetheless, these data have not been previously studied in the setting of concomitant GLP-1RA use, emphasizing a significant gap in the literature.

### 3.2. Exercise

Exercise training has been extensively studied in HF and is a Class I, Level A recommendation in the 2022 ACC/AHA/HFSA guidelines for HF management [40]. Various exercise modalities, including aerobic interval training [41] and cardiac rehabilitation programs [42] have demonstrated benefits in counteracting skeletal muscle wasting, improving functional capacity, and enhancing quality of life in patients with HF [43]. Additionally, these interventions have been shown to increase muscle mass and function while reducing inflammatory markers and oxidative stress [44]. Such effects are particularly relevant in the context of CC, where the multifactorial pathophysiology involves neurohormonal activation, chronic inflammation, and metabolic imbalances. While the evidence supporting exercise training in HF is robust, most studies and meta-analyses have included the general HF population without specifically targeting patients with CC or those treated with GLP-1Ras [40,43]. Nevertheless, given its proposed benefits, exercise training remains a cornerstone of nonpharmacologic management for sarcopenia-mitigating measures in patients with HF.

### 3.3. Pharmacologic Interventions

Pharmacologic treatments for CC or sarcopenia prevention aim to address its complex pathophysiology, which includes neurohormonal abnormalities, immune activation, metabolic hormonal imbalances, and gastrointestinal dysfunction (Table 3). Sympathetic nervous system overactivation is a key driver of weight loss in CC. The COPERNICUS trial demonstrated that carvedilol mitigated cachexia in patients with severe chronic HF (LVEF < 25%). In this randomized, double-blind trial of 2289 patients, those in the carvedilol group were 33% less likely to experience significant weight loss (>6%) and 37% more likely to gain significant weight (≥5%) compared to the placebo [45]. The SOLVD trial, an observational study of 1929 patients with chronic HF, found that enalapril reduced the hazard of significant weight loss (≥6%) by 19% compared to non-users, emphasizing the potential role of renin–angiotensin–aldosterone system inhibitors in mitigating cachexia [46].

Given the multifactorial nature of CC and sarcopenia in HF, additional therapeutic agents have been investigated, including anabolic agents, appetite stimulants, beta-2 agonists, and anti-inflammatory drugs. Testosterone therapy has demonstrated benefits in multiple trials, improving exercise capacity and muscle strength in HF patients [47,48,49]. In combination with exercise, testosterone has been shown to increase lean muscle mass and muscle fiber cross-sectional area [50]; however, its impact on overall cardiovascular health remains an area of contention. Ghrelin and ghrelin agonists, which enhance appetite and nutritional intake, have shown efficacy in increasing lean body mass and overall weight, suggesting their potential in managing CC [51].

Agents such as salbutamol and clenbuterol have shown increases in muscle mass. However, their lack of improvement in left ventricular ejection fraction (LVEF) and the concerning reduction in exercise duration with clenbuterol treatment highlight their limitations [52,53]. Celecoxib has shown benefits in cancer-related cachexia, improving weight, quality of life, and grip strength [54,55,56]. While no studies have directly evaluated celecoxib in CC, its potential role in this setting remains a gap in the literature [57]. Anti-TNF agents such as etanercept and infliximab have been tested in HF but did not show significant benefit [58,59]. Thalidomide, though not studied for weight outcomes, has shown a reduction in TNF-α levels and improvement in ejection fraction, suggesting anti-inflammatory benefits [60], which may be extrapolated to potential benefit in patients with CC.

Several novel pharmacologic interventions for sarcopenia and cachexia are currently under investigation [61,62,63,64]. Enobosarm has shown potential in increasing muscle mass and strength, though its impact on physical function remains limited [61,62,63,64]. Myostatin inhibitors and activin Type II receptor antagonists target muscle growth pathways and are being explored for their ability to enhance muscle function. Bermekimab and bimagrumab target muscle-specific metabolic pathways, while ponsegromab, a growth differentiation factor-15 (GDF15) inhibitor, is a promising candidate currently in phase II trials [61,63,65].

Adjunct therapies targeted at specific pathophysiologic mechanisms related to sarcopenia and CC prevention are critical. However, further studies are needed to establish definitive treatment protocols tailored to the unique needs of patients with advanced HF, particularly in those on GLP-1RAs.

## 4. Gaps in the Literature on Cardiac Cachexia

Several critical gaps remain in the understanding and management of HF-related CC and sarcopenia while on GLP-1RAs (Table 4). These include the absence of standardized diagnostic criteria, limited insights into its pathophysiological mechanisms, and a lack of clinical trials specifically evaluating therapeutic options for this condition. Current treatment strategies are often extrapolated from general HF management, emphasizing the urgent need for targeted therapies that address the unique aspects of CC and sarcopenia [44,66].

With the increasing use of GLP-1RAs to improve cardiovascular risk factors, there is an urgent need to assess their safety and efficacy in patients with CC. At present, it is unclear whether the benefits observed with GLP-1RAs in broader populations are generalizable to this vulnerable group. Rigorous research, spanning preclinical studies to large-scale randomized controlled trials, are essential to determine whether GLP-1RAs can or should be incorporated into the management of advanced HF in those with known CC [67].

Another critical gap is the lack of evidence regarding interventions beyond guideline-directed medical therapy for HF that could prevent the development of CC. While nutritional support and exercise programs have shown promise and are recommended as part of holistic management, additional research is required to develop and validate specific preventive strategies and pharmacologic therapies tailored to the distinct needs of patients with CC [33].

## 5. Conclusions

Cardiac cachexia and sarcopenia are critical yet underappreciated complications of advanced HF, with significant gaps in understanding their pathophysiology and management. The use of GLP-1RAs in this population may exacerbate these conditions, highlighting the need for additional sarcopenia-mitigating measures. While targeted interventions are often necessary, the optimal strategies for preventing muscle loss in patients with advanced HF on GLP-1RAs remain unclear.

## Figures and Tables

**Table 1:** Body composition loss. Types of body composition loss associated with GLP-1RAs.

Therapy	Study Duration	% Reduction in Fat Mass (FM)	% Reduction in Lean Mass (LM)	Key Findings
Semaglutide	68 weeks	24.7%	13.9%	Significant weight reduction but concurrent muscle loss
Tirzepatide	72 weeks	33.9%	10.9%	Higher FM reduction but also notable LM loss

Abbreviations: FM = fat mass, GLP-1RA = glucagon-like peptide-1 receptor agonists, LM = lean mass.

**Table 2:** Nutritional support. Trial data regarding potential nutritional advantages in populations with heart failure.

Intervention	Study Design	Population	Key Outcomes
High-calorie, high-protein supplement	Clinical trial, 18 weeks	HF patients	↑ Body weight, ↓ TNF-α, ↑ Quality of life
Slow-release carbohydrates, fiber, fatty acids	Clinical trial, 6 months	HF patients	↑ Body weight, ↓ Inflammatory markers
L-alanyl-L-glutamine + PUFAs	Clinical trial, 3 months	HF patients	↑ Lean body mass, ↑ Quality of life

Abbreviations: HF = heart failure, PUFA = polyunsaturated fatty acids.

**Table 3:** Pharmacological support. Body composition modulation with medication use.

Therapy	Target Mechanism	Key Outcomes	Limitations
Carvedilol	SNS overactivation	Reduced cachexia prevalence	Not specific to cachexia
Enalapril	RAAS modulation	Reduced significant weight loss hazard (19%)	General HF benefit, not specific
Testosterone therapy	Anabolic agent	↑ Exercise capacity, ↑ Muscle strength	Cardiovascular health concerns
Ghrelin agonists	Appetite enhancement	↑ Lean body mass	Limited HF-specific data
Celecoxib	Anti-inflammatory	↑ Quality of life	Lack of direct HF cachexia trials

Abbreviations: HF = heart failure, SNS = sympathetic nervous system, RAAS = renin–angiotensin–aldosterone system.

**Table 4:** Research gaps and limitations.

Research Gap	Current Limitations	Future Directions
Lack of standardized diagnostic criteria	Heterogeneity in clinical trials	Develop and validate unified criteria
Limited studies on GLP-1 agonists	Uncertain effects on muscle wasting in HF patients	Conduct HF-specific RCTs on sarcopenia mitigation
Preventive strategies for CC	Reliance on general HF guidelines	Tailor strategies specific to patients with cachexia

Abbreviations: CC = cardiac cachexia, HF = heart failure.
